# Bioinformatics-based Investigation to Unveiling The miRNA-Immunity Axis in The Tumor Microenvironment of Pancreatic Cancer

**DOI:** 10.12688/f1000research.172407.2

**Published:** 2026-03-05

**Authors:** Mohammed Salih AL-Janaby, Salah H. Mannoush, Saif S. Al-Janabi, Ahmed AbdulJabbar Suleiman, Ali Hazim Abdulkareem, Anmar Kamil Alalwani, Mustafa M. Fahad

**Affiliations:** 1 Department of biotechnology, College of science, University of Anbar, Al-Anbar, Al_Ramadi, 31001, Iraq; 2 Center of Desert Studies, University of Anbar, Al-Anbar, Al_Ramadi, 31001, Iraq

**Keywords:** Pancreatic cancer, immunotherapy, tumor microenvironment, immune suppression, Memory CD4+ T-cell modulation

## Abstract

**Background:**

In this Bioinformatics study, we took in account the cell types variation of tumor microenvironment (TME) in Pancreatic Ductal Adenocarcinoma (PDAC), with a particular attention to miRNAs dysregulated pathways influencing immune responses, and especially memory CD4+ T cells.

**Methods:**

For cell types and differentially expressed genes in memory CD4+ T cells, scRNA-seq was conducted on PDAC and non-tumoral pancreas samples. Putative miRNA targeting sequences on downregulated genes were investigated by three prediction databases, MiRWalk, TargetScan, and miRDB, and compared to GSE207345’s differential expression with creation of Affymetrix Multispecies miRNA-4 Array. The miRNA–mRNA interaction network was established in Cytoscape.

**Results:**

Memory CD4+ T cells were significantly enriched in PDAC TME. At the same time, we observed genotype-specific repression of major immune and metabolic genes in these cells. Some miRNAs were significantly overexpressed in PDAC, and hsa-miR-1207-5p, hsa-miR-6805-5p, and hsa-miR-149-3p might target many immune-related or pancreatic enzyme-associated genes. Of these, a core of *CLPS*, *NR4A2*, and *SOCS3* was most frequently targeted, which likely indicated the importance of these genes in immune evasion.

**Conclusions:**

While memory CD4+ T cells exist within the PDAC TME, their activity is suppressed by miRNA-associated gene dysregulation. These data suggest candidate therapeutic approaches in which targeting of miRNAs could be used to normalize immune cells and promote anti-tumor immunity.

## Introduction

PDAC, more than 90% of pancreatic cancer cases, possesses a less than 10% survival rate at five years due to late clinical presentations and fast progression. Despite the fact that it accounts for just 2% of cancer altogether, pancreatic cancer contributes to 5% of all U. S. cancer deaths and an annualized increase in its fatality rate at approximately 1%. PDAC is estimated to be the 2nd most fatal cancer by 2030. The surgical technique should be based on the tumor stage: surgery cures for non-metastasized cases, however not more than 20% of patients can receive radical intervention due to the advanced stage and relapses. ^1^

The PDAC TME is known to be highly immunosuppressive and non-immunogenic, presenting a major challenge for elicitation of successful immune responses. It comprises of the tumor cells, stromal support cells, immune cells and dense extracellular matrix. Desmoplasia, a fibrosis-like matrix deposition wherein stromal-tumor crosstalk directs aberrant tissue structure, is a hallmark of PDAC, contributing to hypoxia, progression and immune tolerance. ^2– 4^

Even that PDAC tissues are presented with CD4 ^+^ and CD8 ^+^ T cells, most of them are inactivated by TME-induced immunosuppression. Although increased T-cell levels are associated with improved survival in certain scenarios, ^5^ the TME immobilizes their efficacy. Inhibitory constituencies, such as T reg cells, myeloid-derived suppressor cells (MDSCs), and tumor-associated macrophages produce inhibitory molecules including TGF-β, IL-6, and ARG-1, ^6^ which also inhibit T-cell activation. Furthermore, cancer associated fibroblasts facilitate evasion of the immune response, angiogenesis and metastasis. These mechanisms disable infiltrating T cells and promote PDAC’s immune resistance. ^7^ Dysregulation of microRNA (miRNA) in T cells ^8^ can modulate anti-tumor immunity. ^9^ MiRNAs are a family of small non-coding RNAs that control more than 60% of protein- coding genes, which include many genes related to immune response. ^10^ Bird, Springer and colleagues talked in language that was even more humanlike: “With the genetics of CD4+ T cells finally decoded, we would have a set list with which to disrupt activation/differentiation/function by modulating gene expression.” OncomiRs including miR-155 and miR-21, overexpressed in PDAC, have been shown to target genes involved in cytokine signaling and immune synapse formation. ^11^ miR-21 induces PI3K/AKT/mTOR signaling which enhances tumor cell survival and chemoresistance, while miR-146a targets NF-kB, thereby disturbing T-cell activation. MiRNAs control the ratio of pro-inflammatory T cells and regulatory T-cells (Tregs), and thus, play an essential role in immune suppression in PDAC. Clusters like miR-10a and miR-17-92 favour Treg differentiation and suppress CD8 ^+^ T cell function. miR-29b and miR-155 have also been reported to modulate immune checkpoint pathways, leading to an impairment of T-cell activity. miR-155 promotes immune evasion through targeting *SOCS1* to activate *STAT3*, thereby mediating MDSC expansion. ^12^ The immunosuppressive profile of PDAC is improved by miR-7b-5p which facilitates glycolysis-mediated immune evasion. Moreover, miR-10a-5p, ^7, 13^ which is overexpressed in gemcitabine-resistant PDAC, promotes tumor migration and invasion. miR-17-5p regulates tumorigenesis and chemosensitivity through RBL2. ^14^

The research explores the differences at a cellular level between the tumor microenvironment (TME) of PDAC and non-tumor tissue by using single-cell RNA sequencing (scRNA-seq). It pinpoints immune subtypes and downregulated markers, which are overlapped with dysregulated miRNAs in microarray. This analysis reveals cell type-specific miRNA-mRNA networks that further our understanding of immune regulation in PDAC at the cellular level.

## Methods

### Ethical considerations

This study utilized publicly available data from the National Center for Biotechnology Information (NCBI) Gene Expression Omnibus (GEO) ( https://www.ncbi.nlm.nih.gov/gds), a platform for high-throughput genomics and gene expression datasets. Data usage complied with the original researchers’ guidelines on sharing, privacy, and publication.

### Type of sampling and reasons for selection

A tissue-specific sampling approach was applied using PDAC and matched non-tumor tissues. Sectioned tissue samples enabled detailed analysis of the tumor microenvironment (TME), immune cell populations, dysregulated miRNAs, and metabolic alterations. Focusing on tissue specificity reduced confounding effects common in systemic sampling methods.

### Patient consent statement

This study did not involve human participants directly and therefore did not require informed consent or IRB approval.

### Inclusion criteria

Included datasets featured pancreatic ductal adenocarcinoma (PDAC) and adjacent normal tissues, all with high-quality metadata. Only datasets using single-cell RNA sequencing (scRNA-seq) of PDAC and healthy pancreatic tissues were considered. Additionally, miRNA expression profiles used were either experimentally validated or high-confidence predictions, minimizing error risks and supporting robust analysis of cell-specific regulatory patterns.

### Single-cell RNA-sequencing data processing

Preprocessed scRNA-seq data for PDAC were obtained from the NCBI Gene Expression Omnibus (GEO, https://www.ncbi.nlm.nih.gov/gds), a public database for high-throughput genomic datasets. The dataset GSE155698 includes 41 samples (tissue and PBMC), from which 19 pancreatic tissue samples (16 PDAC, 3 normal) were selected for analysis. Seurat v4.4.0 was used for quality control and downstream processing. ^15^

Low-quality cells were excluded based on gene count, UMI content, and mitochondrial gene percentage. Specifically, cells with >6000 genes (possible doublets), <200 genes (low quality), mitochondrial reads >10%, or nCount_RNA <1000 were filtered out. Normalization was applied using the “LogNormalize” method, followed by data scaling and identification of 2000 highly variable genes using Seurat’s “FindVariableFeatures”. Dimensionality reduction was conducted via t-SNE using the first 20 principal components, and cells were clustered using the Louvain algorithm. Cell type annotation was performed using the scType R package and a validated pancreas marker database ( https://sctype.app/). ^16^ Immune cell subsets were further subclustered to refine resolution and characterize immune diversity within the PDAC tumor microenvironment.

### Cell type proportion and differential expression analysis

To assess differences in cell type proportions between PDAC and healthy pancreatic tissue, percentages were calculated and visualized using ggplot2, with statistical significance determined via t-test using the ggpubr package. ^17^ Memory CD4 ^+^ T cells, showing significant differences in abundance, were selected for further analysis. Differential expression was assessed using the Wilcoxon test; genes with logFC > 0.25 or < -0.25 and p < 0.05 were considered significant. Downregulated marker scores were calculated using AddModuleScore in Seurat, and grouped by cell type for comparison between PDAC and healthy samples.

### Prediction of miRNA targets of immunological markers

Downregulated genes in Memory CD4 ^+^ T cells were analyzed for potential miRNA targets using three tools: MiRWalk **(** http://mirwalk.umm.uni-heidelberg.de/ **)**, TargetScan **(** https://www.targetscan.org/vert_80/ **)**, and miRDB **(** https://mirdb.org/ **)**. These tools predict miRNA-mRNA interactions based on conservation, experimental validation, or high-throughput data. ^18^ Predicted interactions were filtered using pandas in Python, retaining miRNAs with binding probability = 1, energy score ≤ -25, and AU content < 0.6 in the 30-nucleotide flanking regions. De novo miRNAs predicted by MiRWalk were also included. The final shortlist comprised miRNAs meeting all criteria across tools for further downstream analysis.

### MiRNA differential expression analysis in PDAC tumor

MicroRNAs (miRNAs) exhibit tissue-specific expression patterns and are involved in gene regulation under both normal and pathological conditions. To identify dysregulated miRNAs in pancreatic ductal adenocarcinoma (PDAC) relative to normal pancreatic tissue, differential expression analysis was conducted using GEO2R ( https://www.ncbi.nlm.nih.gov/geo/geo2r) on dataset GSE207345, which includes 7 PDAC tumor samples and 6 adjacent normal tissue samples. This dataset was generated using the Affymetrix Multispecies miRNA-4 Array. miRNAs with a p-value < 0.05 and a log fold change (logFC) < -1.0 or > 1.0 were classified as significantly dysregulated and selected for further evaluation.

To integrate this miRNA analysis with transcriptomic findings from single-cell RNA sequencing (scRNA-seq), we focused on genes that were downregulated in memory CD4 ^+^ T cells from PDAC samples. These were cross-referenced with the list of upregulated miRNAs identified in the tumor dataset. Only miRNAs that were both upregulated and predicted to target these downregulated genes were retained, highlighting their possible roles in suppressing immune functions in the tumor microenvironment.

### MiRNA-mRNA network analysis

We used Cytoscape ( https://cytoscape.org/) to construct and visualize miRNA-mRNA interaction networks. ^19^ Each miRNA and its target genes were represented as nodes connected by interaction edges. This visualization helped identify key regulatory miRNAs. Cytoscape also provided network analysis tools to detect highly connected nodes and functional modules, enhancing interpretation of miRNA regulatory roles in PDAC pathogenesis. ^20^

### Statistical analysis

Multiple statistical methods were applied in this study. Cell type proportions in PDAC and healthy tissues were compared using t-tests and visualized with ggplot2. Memory CD4 ^+^ T cells were analyzed via Wilcoxon test, considering genes with logFC > 0.25 or < -0.25 and p < 0.05 as significant. Seurat’s AddModuleScore function was used to assess downregulated markers, with results visualized using ggpubr. Differentially expressed miRNAs were identified via GEO2R, using thresholds of logFC < -1.0 or > 1.0 and p < 0.05. miRNA-mRNA interactions were predicted using MiRWalk, TargetScan, and miRDB, filtered based on binding score, energy, and AU content. Network visualization was performed using Cytoscape.

## Results

### Distinct cellular composition and immune cell enrichment in the PDAC tumor microenvironment

**Figure 1.**
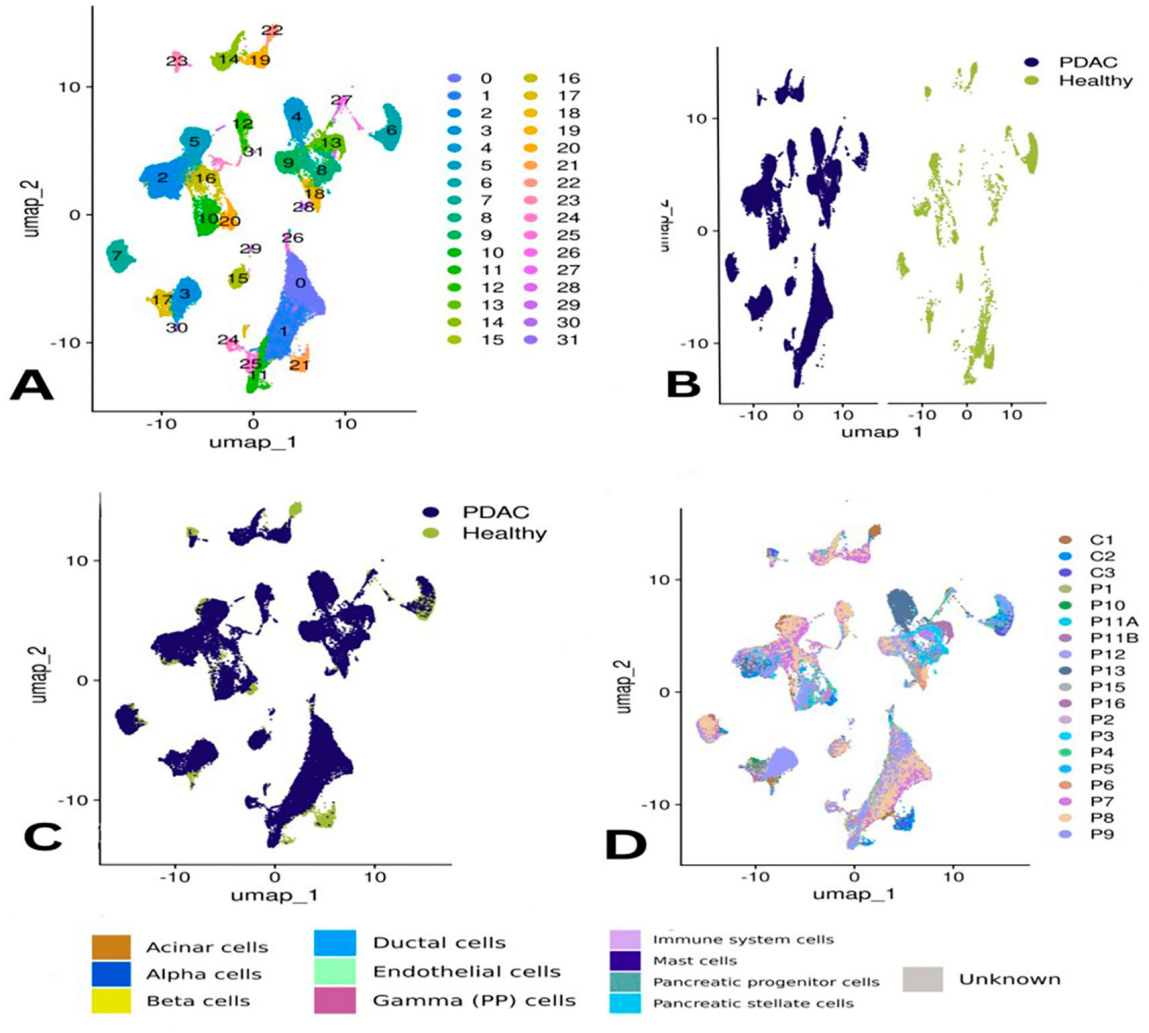
Illustrates the overall clustering of cells in PDAC and healthy samples using UMAP. Panel (A) shows the clusters based on gene expression profiles, while (B) and (C) compare clustering by condition—PDAC (chocolate) vs. healthy (teal). Panel (D) visualizes clusters by donor sample ID.

**Figure 2.**
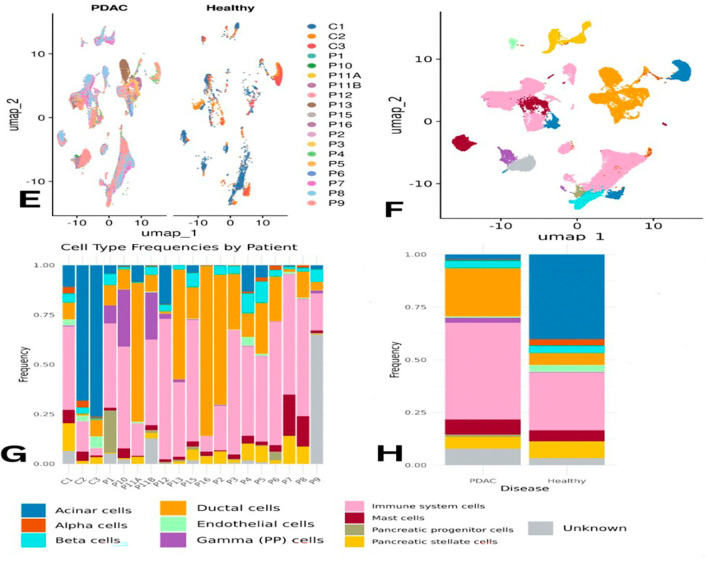
Expands on donor-based clustering and cell type annotation. Panel (E) depicts cluster identity per donor, and (F) annotates each cluster by specific pancreatic cell types. Panel (G) presents differences in the proportions of these cell types across all samples, and (H) compares abundances by condition, highlighting the shift in cellular composition in PDAC.

The PDAC scRNA-seq dataset initially includ ed32,738 RNA features and 107,298 cells. Following quality control, 71,232 high-quality cells were retained by removing dead cells and those with abnormal gene profiles. Using highly variable genes and dimensionality reduction methods, a total of 31 distinct clusters were identified and annotated ( Figure 1A). Most clusters were broadly represented across samples, though clusters 22, 30, 29, 23, 6, 11, and 21 were predominantly composed of cells from healthy tissues ( Figure 1B–C). Additionally, cluster 4 was found to consist entirely of cells from sample P13 ( Figure 2E).

Pancreatic cell types were identified, including acinar, alpha, beta, ductal, gamma (PP), mast cell, stellate cells and progenitor and immune cells ( Figure 2F). Of interest, the unidentified cells comprised a greater portion (65%) of patient P9 and all other subpopulation were less represented including mast, ductal and acinar cells, immune cells accounted for (20%) similar to in their sample. Similar to P2, small proportions of CREB3L3-expressing cells were also present in other samples (<5%), with the exception of P11B showing higher proportion (~18%) ( Figure 2G).

Acinar cells were the predominant cell type in controls C2 and C3 at approximately 75%, while that of C1 was substantially less (15%). Ductal and immune cells were well-represented in control and PDAC samples. Gamma (PP) cells were observed in samples P1, P10, and P11B. Of interest, specimen P16rapresented the highest concentration of ductal ceIIs as achieved in specimen_P11A. Mast cells were identified in every sample, to different degrees.

### Immune cell distribution in PDAC tumor microenvironment

**Figure 3.**
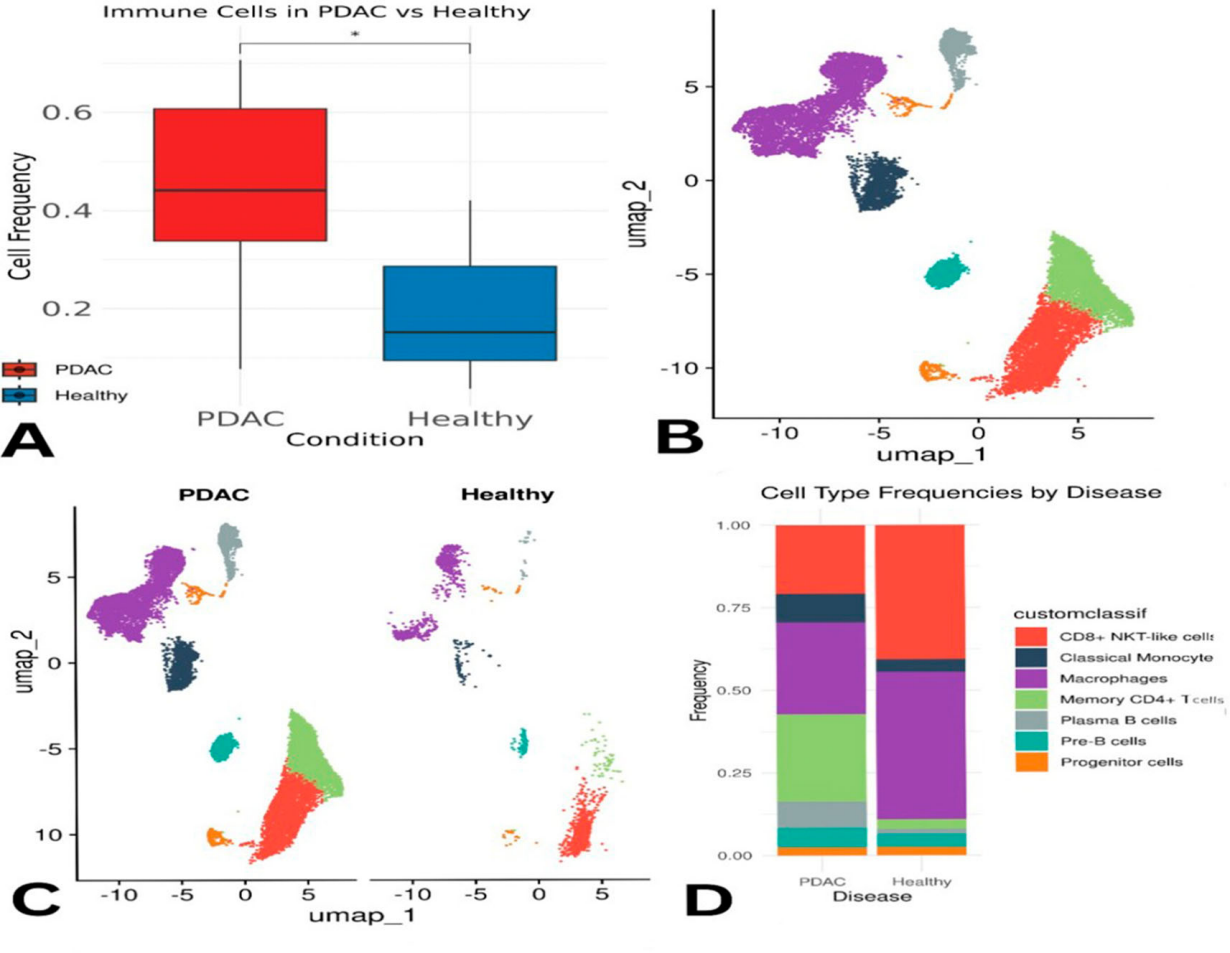
Focuses on immune cell enrichment. Panel (A) shows statistical analysis of immune cell abundance between groups. Panels (B) and (C) visualize immune-specific clusters by condition. Panel (D) details shifts in proportions of individual immune cell types between PDAC and healthy tissues.

When comparing PDAC and healthy samples collectively, immune cells were the most abundant in PDAC, accounting for approximately 45% of the total population versus ~20% in healthy tissue. Conversely, acinar cells were more frequent in healthy samples (~35%) but dropped to ~2% in PDAC. Ductal cells increased in PDAC (~25%) compared to ~5% in controls ( Figure 2H). The distribution of some cell types was uneven across individual samples ( Figure 2G). Statistical analysis confirmed that immune cells were significantly more prevalent in PDAC, underlining their central role in the tumor microenvironment ( Figure 3A).

### Differential abundance and sub classification of immune cells in PDAC tumor microenvironment

Due to the statistically significant difference in immune cell abundance between PDAC and healthy samples, immune system cells were subsetted and subclustered to identify their subtypes. Using the scType database with immune markers, the following cell types were annotated: CD8 ^+^ NKT-like cells, Classical Monocytes, Macrophages, Memory CD4 ^+^ T cells, Plasma B cells, Pre-B cells, and Progenitor cells ( Figure 3B).

**Figure 4.**
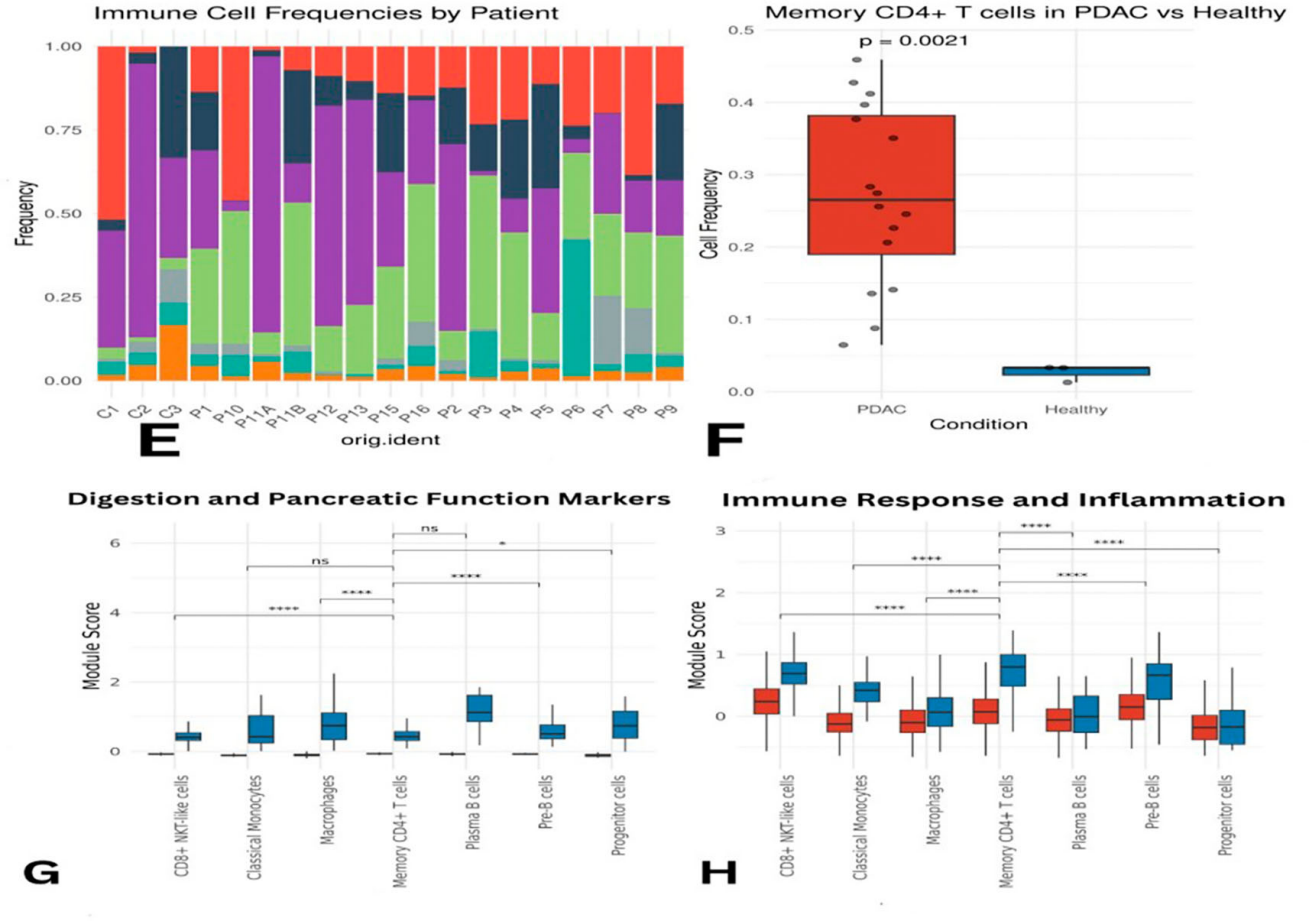
Breaks down immune subpopulations. Panel (E) illustrates differences in abundance across all immune cell types, while (F) presents statistical evidence of elevated memory CD4+ T cells in PDAC. Panels (G) and (H) display module scores showing reduced expression of digestion- and immune-related genes in PDAC memory CD4+ T cells.

Overall, macrophages, plasma B cells, classical monocytes, pre-B cells, CD8 ^+^ NKT-like cells, and memory CD4 ^+^ T cells were found at higher proportions in PDAC than in healthy samples ( Figure 3C–D). However, this might partially reflect the lower number of healthy controls. Memory CD4 ^+^ T cells were slightly more abundant in control samples individually, but collectively more enriched in PDAC. Macrophages appeared across all samples, except for P10 and P6, where they were below 5%. CD8 ^+^ NKT-like cells were present in all PDAC samples (5–35%) and in only one healthy sample (C1) at a concentration of ~50% ( Figure 4E). A Wilcoxon test confirmed that memory CD4 ^+^ T cells were significantly more abundant in PDAC samples, with a p-value of 0.0021 ( Figure 4F).

### Impact of PDAC on memory CD4+ T cells reveals key alterations in immune and metabolic gene expression

The effects of PDAC on memory CD4 ^+^ T cells was detected by differential expression analysis using the Wilcoxon test in Seurat. D. The analysis was successful in finding 4 upregulated genes and 115 downregulated genes. Among them, *RPS26*, *RPS20*, *HBA2* and *HBB* were up-regulated. These genes are not only involved in ribosomal function, but also in oxygen transport underlining higher protein biosynthesis and modified redox processes in PDAC.

The increased expression levels of *RPS26* and *RPS20* could suggest an increase in the protein synthesis capacity as a putative nutrient metabolic adaptation to the tumor microenvironment. In addition, *HBA2* and *HBB*, which also contribute to hemoglobin production, could indicate a dysregulation in oxygen transportation and the tumor hypoxic state response.

Some of the pancreatic enzyme genes such as *PNLIP*, *PRSS1/PRSS2, AMY2A, CLPS, CELA 2A, CPA1, PLA2G1B, CELA3B* and *CTRC* were markedly repressed. These are genes that usually aid in releasing digestive enzymes and their inhibition indicates exocrine dysfunction in PDAC. In addition, *SPINK1*, a tissue-protector gene of pancreatitis, was also down-regulated. Several immune-regulatory genes such as *SOCS3*, *CD69* and *CXCR4* were down regulated that may limit the signaling pathway/transduction of the activation and migration of immune cells. Reduction of *AREG* and *C4BPA*, also known mediators of inflammation and immune regulation, additionally underscores the immuno-suppression characteristic of the PDAC TME.

Transcription regulating genes like *JUN, FOS, NR4A2, HES7* and *JUNB* were downregulated leading to negative regulation of proliferation/survival/stress/inflammation/apoptosis. And the expression levels of metabolic stress response genes, *DUSP1, DDIT4* and *MT1A* were down-regulated. These genes are involved in the adaptation to oxidative stress and regulation of metal ion homeostasis. Module score analysis validated these results, which also exhibited lower expression levels of genes in PDAC than in normal samples ( Figure 4G-H, Figure 3A-B).

### Integrative profiling of miRNA expression and target interactions in PDAC

We identified 18 downregulated miRNAs and 593 upregulated miRNAs by the differential expression analysis of PDAC tumor samples with paired adjacent healthy tissues ( Figure 3C). The previously extracted downregulated genes, and the miRNA targets (strong or weak interactions), were integrated for correlation to predict the miRNA-mRNA interactions. Starting from an initial number of 9,279 interactions (without refined nodes), the set of upregulated miRNAs was maintained and thus the network reduced to 2,094 relationships.

Sequential stringency cut-off was imposed: binding < -25 and AU content < 0.6. This narrowed the list to 1,052 high confidence interactions. Ten miRNAs were hot candidates for downstream analysis: hsa-miR-1207-5p, hsa-miR-6805-5p, hsa-miR-149–3p, hsa-miR-762, hsa-miR-6846–5p, hsamiR-7109-5p, hsami-R-5787, hsa-mi R-6848–5 p, hsa -mi R -197 most of which had logFC values ranging from 1.4 to 1.6 ( Figure 3 D).

Functionally, the miRNAs target major genes responsible for immune suppression, inflammation, tumor advancement as well as stress evasion. For instance, hsa-miR-1207-5p targets with *AREG, CLPS, CTRC, SPINK1* and *SOCS3*; whereas hsa-miR-6805-5p interplays with *FOS, JUN, PNLIP* and *NR4A2* which were all essential for immune signaling. hsa-miR-149-3p also targets CXCR4, SOCS3 and CHAC1, thereby affecting migration and stress regulation. In addition, hsa-miR-762 targets *DDIT4, DUSP1, NR4A2* and other inflammation- and metabolism-associated genes. These data emphasize heterogeneity of the PDAC regulatory landscape, in which induced miRNAs may serve as master regulators controlling immune evasion and tumor resistance. Targeting these miRNAs may potentially provide novel therapeutic approaches to restore immune responses and inhibit the progression of PDAC.

### Functional clustering of miRNAs and their target genes

Additionally, hsa-miR-6846-5p targets *CHAC1, CLPS, CPA1, FOS, HES7, NR4A2, SPINK1*, and *SYCN*—genes involved in cellular stress response, apoptosis, and proliferation. Four other miRNAs—hsa-miR-7109-5p, hsa-miR-5787, hsa-miR-6848-5p, and hsa-miR-197-5p—also regulate genes implicated in immune modulation, inflammatory signaling, and cellular stress, hsa-miR-7109-5p targets *C4BPA, CEL, CHAC1, CLPS, CXCR4, DDIT4, NR4A2*, and *SOCS3*, which are crucial for immune cell migration and signaling. Similarly, hsa-miR-5787 regulates *CLPS, CTRC, DDIT4, FOS, INS, PRSS3*, and *SPINK1*, all of which are important in stress and inflammatory pathways. hsa-miR-6848-5p is predicted to target *AREG, BTG1, CTRB2, INS, NR4A2, REG1A*, and *SPINK1*, while hsa-miR-197-5p interacts with *CHAC1, CPA1, GP2, INS, JUNB*, and *SYCN*—genes related to immune cell behavior and oxidative stress responses. These interactions are detailed in Table 1.

**Table 1.** Top upregulated miRNAs in PDAC and their target interactions.

miRNA ID	Interactions	Genes
hsa-miR-1207-5p	9	*AREG, CLPS, CTRB2, CTRC, HES7, JUNB, NR4A2, PRSS3, SPINK1*
hsa-miR-6805-5p	9	*CLPS, FOS, JUN, JUNB, NR4A2, PNLIP, PRSS3, SOCS3, SYCN*
hsa-miR-149-3p	8	*BTG1, CHAC1, CTRB1, CTRB2, CXCR4, HES7, SOCS3, SYCN*
hsa-miR-762	8	*CLPS, CTRC, DDIT4, DUSP1, GP2, INS, NR4A2, PRSS3*
hsa-miR-6846-5p	8	*CHAC1, CLPS, CPA1, FOS, HES7, NR4A2, SPINK1, SYCN*
hsa-miR-7109-5p	8	*C4BPA, CEL, CHAC1, CLPS, CXCR4, DDIT4, NR4A2, SOCS3*
hsa-miR-5787	7	*CLPS, CTRC, DDIT4, FOS, INS, PRSS3, SPINK1*
hsa-miR-6848-5p	7	*AREG, BTG1, CTRB2, INS, NR4A2, REG1A, SPINK1*
hsa-miR-197-5p	6	*CHAC1, CPA1, GP2, INS, JUNB, SYCN*
hsa-miR-6778-5p	6	*CTRB2, CTRC, DDIT4, HES7, JUNB, SOCS3*

Interestingly, many miRNAs converge on the same targets, emphasizing their central regulatory roles. For example, *CLPS, NR4A2, PRSS3, SPINK1*, and *CTRC* are common targets of multiple miRNAs, underlining their involvement in inflammation, immune signaling, and enzyme regulation. *CLPS* is co-targeted by hsa-miR-1207-5p, hsa-miR-6805-5p, and hsa-miR-762, while *NR4A2* is regulated by hsa-miR-6805-5p, hsa-miR-762, and hsa-miR-7109-5p. *PRSS3* and *SPINK1* are also recurrent targets of several immune-modulating miRNAs such as hsa-miR-1207-5p, hsa-miR-5787, and hsa-miR-7109-5p. Figure 3E illustrates this overlap.

The top upregulated miRNAs in PDAC and their predicted target interactions are summarized in Table 1.

The overall clustering of PDAC and healthy cells was visualized using UMAP ( Figure 1).

Donor-based clustering and pancreatic cell type annotation are shown in ( Figure 2).

Immune cell enrichment between PDAC and healthy tissues is presented in ( Figure 3).

Differences in immune subpopulations, particularly elevated memory CD4+ T cells, are highlighted in ( Figure 4).

**Figure 5.**
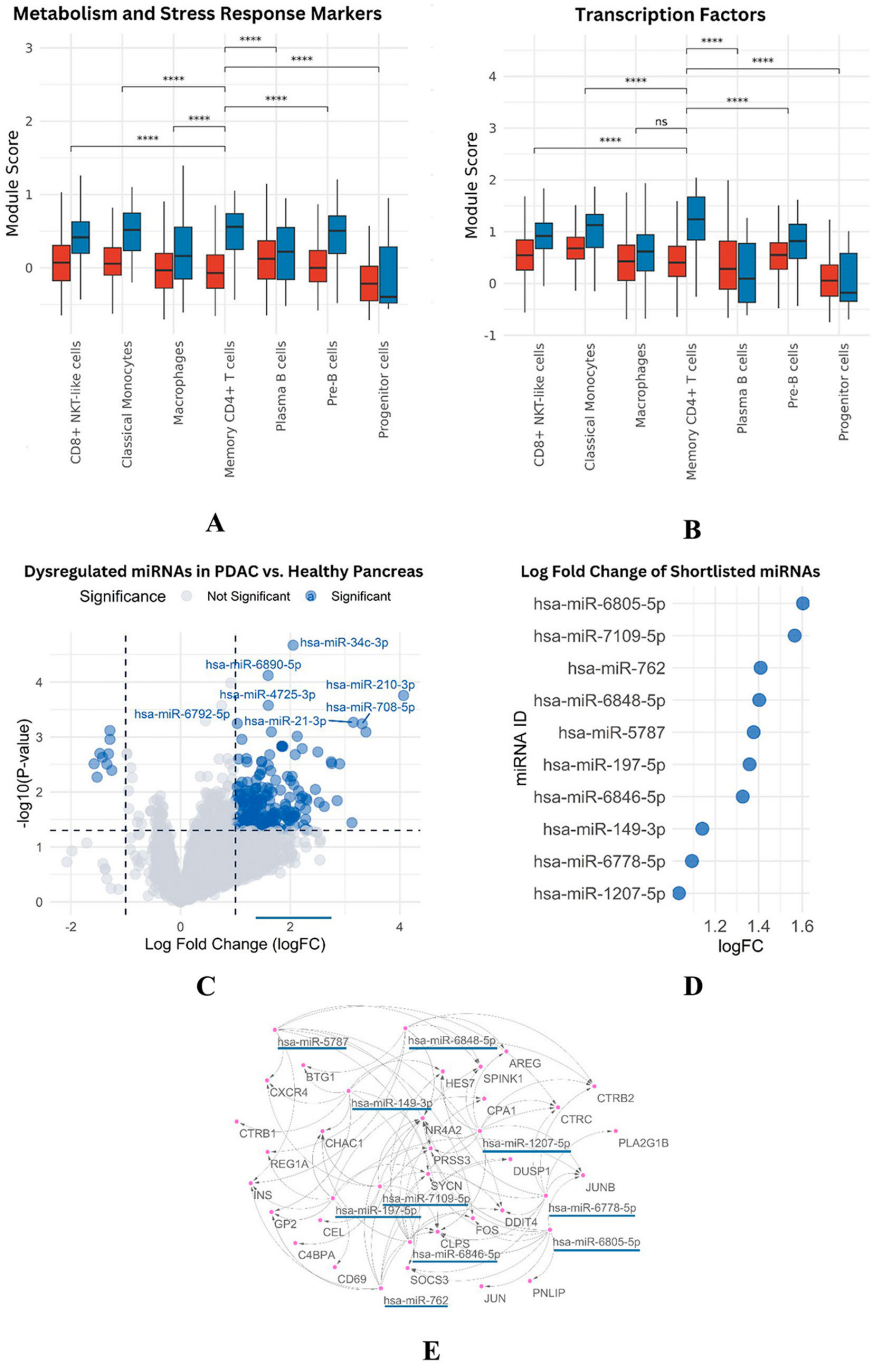
Examines miRNA effects. Panels (A-B) show reduced metabolic and transcription factor activity in PDAC memory CD4+ T cells. Panel (C) presents a volcano plot of differentially expressed miRNAs. Panel (D) lists significantly upregulated miRNAs with high logFC values, and (E) displays the interaction network between miRNAs and downregulated mRNA targets.

The effects of miRNAs, including a volcano plot and interaction network, are illustrated in ( Figure 5).

## Discussion

This article presents a powerful cellular roadmap of the PDAC TME, with many cell subtypes and interactions. Interestingly, immune cells also account for a prominent fraction of PDAC tissue mass and are believed to have an important effect on the TME, making them ideal targets for drug therapy. ^4^ Previous reports support the predominance of immune cells in PDAC, which can induce tumor progression by driving immunosuppression, extracellular matrix (ECM) remodeling and cytokine production. In addition, T cell expression of PD-1 and TIGIT ^16^ is associated with a state of T cell dysfunction, for which dual checkpoint blockade has been suggested as an attractive therapy approach. ^21^

A dramatic loss of acinar cells in the samples derived from PDAC was also observed, suggesting massive tissue reorganization. In contrast, an increased proportion of ductal cells could be a compensatory or pathologic response to tumor development. The diversity of cell-type proportions among the individuals highlights the complex and personalized nature of PDAC, which requires individual therapy. ^22^ The progression of PDAC is characterized by acinar-to-ductal metaplasia (ADM) that results in transformation of acinar cells into duct-like cells and, which are induced by KRAS mutations. This conversion aids in the development of fibrotic stroma and assists in immune cell exclusion by mixed specialization of physical blockade with hypoxia. The derecruitment of acinar identity and increase in ductal cells distorts the immune homeostasis allowing the recruitment of regulatory T cells (Tregs) and M2 macrophages, which further promotes tumor progression and immune evasion. ^23^

PDAC samples were enriched for memory CD4 + T cells. This suggests that active immune infiltrate at superficial levels -observed by our initial digital quantification- is paradoxically in parallel with very extensive immune failure, with global immunometabolic gene suppression. The existence of these cells may be a recruited inflammatory, but with its disables a function it involves blocks the tumor suppression mechanisms and is working. ^24^ Cellular heterogeneity is one of the major characteristics of PDAC where multiple populations of cancer, immune and stromal cells interact with each other that limit the treatment modalities. Several targeted therapies are now focused on reprogramming macrophage polarization, reversing T cell exhaustion and altering tumor vasculature and for precision medicine strategies to profile the microbiome. ^25, 26^ Metabolic dysfunction seems to be critical. For instance, genes like *RPS26* and *RPS20* are upregulated in memory CD4+ T cells which may be a sign of enhanced protein synthesis due to tumor related metabolic stress. In the same vein, increased levels of HBA2 and HBB might reflect adaptation to hypoxic tumor microenvironment. ^25^ Nevertheless, this adaptation might also lead to immune exhaustion and thus to decreased capability of those cells to mediate an anti-tumor reaction. ^27^

These results highlight the fact that while memory CD4+ T cells are abundant in PDAC, their potential functionality is hampered because of metabolic rewiring and immune suppression in tumor stroma.

The down-regulation of *SOCS3, CD69* and *CXCR4* suggests that signaling, pinpoints at inefficient stimulation of the memory cells resulting in impeded activation and migration of the memory CD4 T-cells. ^28, 29^ Key to maintain tissue integrity and immune suppression such as *SPINK1* and *AREG* are also highly down-regulated, attenuating PDAC’s defense system. ^30, 31^ With their expansion, however, such T cells become hypofunctional and are thought to contribute to tumor immune evasion as well as progression. ^29^ Global miRNA profiling of PDAC identified 593 upregulated versus 18 downregulated miRNAs, indicating wide-ranging post-transcriptional regulation in support of a repressive immune response. For example, hsa-miR-1207-5p targets *SOCS3, SPINK1* and *AREG*, which interferes with pathways that are crucial for immunosuppression regulation and tumor progression. hsa-miR-6805-5p also targets *CXCR4, SOCS3*, and *JUN*, known to control migration and signaling of immune cells.

Furthermore, hsa-miR-149-3p also targets *CHAC1, CXCR4* and *SOCS3* leading to an immune cell homing and stress response defect. ^32^ Convergence of number of miRNAs upon critical immune genes demonstrates a potent regulation mechanism behind immune repression in PDAC. ^33^ In particular, *SOCS3*, a negative regulator of the JAK/STAT pathway is targeted by several miRNAs and among them by miRNA-203 to enhance STAT3 activity and contribute to immune evasion. ^34, 35^

The persistent downregulation of *SOCS3* and immune checkpoint disruption indicate that miRNA targeting of immune genes is a key mechanism underlying the immune-evasive phenotype observed in PDAC. CD69, a surface marker of early activation on T cells, commonly suggests vigorous immune activation. Yet, in pancreatic ductal adenocarcinoma (PDAC), the coexpression of LAG3 with inhibitory checkpoint receptors as PD-1 and TIGIT indicates a landscape of T cell exhaustion suggesting a multifaceted abnormal regulation of immunity. Reduced expression of *CXCR4*, which is essential for recruitment of T cells, may also hinder the homing process of immune cells to the tumor microenvironment, compromising immune surveillance and suppressing anti-tumor immune response. ^36^

This suppression is also supported by the upregulation of miRNA in PDAC like hsa-miR-762, hsa-miR-7109-5p and hsamiR197-5p that act through direct binding to key target genes related with stress response mechanism, transcriptional regulation ^37^ and immune activating pathways. ^38^ These miRNAs go against key genes “modulators of immune cell adjustment for stress (e.g., *DUSP1, DDIT4* and *NR4A2*)” responsible for the capacitating memory adaptability and functionality of CD4 T cells under in tumor conditions. ^39^ Transcriptional factors JUN, JUNB and FOS that are critical for T-cell expansion, differentiation and activation also appear to be highly inhibited. The down-modulation was likely reflecting tumor-mediated T cell effector dysfunction and closely resembled the immune exhausted phenotype. Although their specific roles in PDAC-memory CD4+ T cells remain poorly characterized, the inhibition of these adaptations highlights a broader failure in metabolic adjustments related to tumor-induced stress. ^24^ In addition, the immune cells present in the TME suffer from severe metabolic limitations in PDAC. Downregulation of regulators of oxidative stress and mTOR signaling, MT1A and DDIT4, is indicative for the loss in metabolic adaptation to suppression and this indeed has a profound effect on the suppressed capacity of these T cells.

Strategies to overcome immune suppression in pancreatic ductal adenocarcinoma (PDAC) are likely to be greatly enhanced by restoring the function of memory CD4+ T cells. ^40^ Improving their immune competence might boost anti-tumor responses. One functional consequence of inhibiting the upregulated miRNAs, such as hsa-miR-1207-5p and hsa-miR-6805-5p, will be to alleviate repression on key immune-regulatory genes (e.g., *SOCS3, NR4A2*, or *SPINK1-like*), which in-turn may reactivate immune signaling pathways. This is a strong foundation for the development of miRNA-targeted therapeutics—e.g., antagomiRs or miRNA sponges—to rescue immune cell function. Such approaches could be combined with the existing immunotherapies to “re-tune” the complicated immunosuppressive milieu of PDAC tumor microenvironment.

This work illustrates a comprehensive cellular/molecular portrait of the PDAC TME, highlighting altered miRNA-target gene interaction in memory CD4+ TIL. Nonetheless, even though the links between dysregulated miRNA profiles and disruption of immunity are well-documented, causality has not been verified.

The miRNAs have not yet been validated experimentally. The roles of these proteins were deduced from bioinformatics predictions and had not been functionally confirmed. While ChIP-qPCR and pull-down assays can provide preliminary evidence, further validation with PDAC models of miRNA knockdown or overexpression is required to establish their role in immunosuppression and tumorigenesis. The authors concede these shortcomings and pose them as subjects for further research. Such studies will be critical for increasing our understanding of immune regulation in PDAC and informing novel and targeted therapies.

Single-cell RNA sequencing (scRNA-seq) is critical in dissecting cellular heterogeneity, however batch effects and technical noise may debilitate the accurate comparison of differentially expressed genes and miRNA-mRNA interaction analysis. While substandard cells were removed and normalization was performed, there may be some existing batch effects, caused largely by the use of public data. These challenges might be addressed in future studies by using more sophisticated tools, such as Harmony or Seurat’s integration functions. To increase the clinical interpretation of miRNAs, we should integrate Transcriptomic findings together with patient outcomes: survival time, progression of tumour, or patients’ response to immunotherapies. For example, it is possible to further enhance the predictive ability by evaluating whether hsa-miR-1207-5p or hsa-miR-6805-5p can combine with clinical outcomes to predict poor prognosis. However, these datasets are not well-annotated in terms of clinical information, and we cannot obtain such insights. The authors acknowledge this limitation and suggest that future studies will integrate miRNA information with clinical indexes in order to search for biomarkers and facilitate personalized treatments.

## Conclusion

This study provides increased insight into the immunosuppressive mechanisms operating in the tumor microenvironment of pancreatic ductal adenocarcinoma. Although the PDAC TME is found to have memory CD4+ T cells, their functionality is largely compromised by widespread downregulation of key immune and metabolic genes. The downregulation observed is probably orchestrated by the upregulation of specific microRNAs, such as hsa-miR-1207-5p, hsa-miR-6805-5p, and hsa-miR-149-3p, targeting a plethora of genes related to immune regulation, inflammation, and pancreatic enzyme production. Genes like *CLPS, NR4A2*, and *SOCS3*, targeted by several microRNAs, emerge as key players in the immune suppression associated with PDAC. These results suggest that the mechanism of immune evasion in PDAC is rather complex and involves miRNAs playing a central role in the inhibition of the functionality of memory CD4+ T cells, impeding anti-tumor immunity and fostering tumor progression.

### Outcomes of the study

This work revealed profound immune deficiency in the PDAC tumor milieu and identified memory CD4+ T cells as the main target of suppression. This paper confirms profound suppression of immune and metabolic genes in these cells accompanied by upregulated miRNAs hsa-miR-1207-5p and hsa-miR-6805-5p. Promising observations reported are that the study of miRNA-mRNA pairs emphasizes potential avenues to replenish the immunologic capacity in PDAC.

### Rationale of the study

Pancreatic ductal adenocarcinoma is still one of the major causes of cancer death as it is highly invasive, rapidly progressing and has low Immunogenicity. This issue generally lacks an explanation of the mechanism of how miRNAs regulate immune suppression in the context of PDAC tumor microenvironment with regard to memory CD4+ T cells. The analyses of scRNA-seq data with miRNA level information provide a multi-layered picture of immune dysregulation and point to tangible targets for immunomodulation.

### Limitations of the study

Therefore, some variability can arise due to the differences in sample handling and sequencing methodologies from those that were used in this study but originated from public repositories. Real-time exclusion of systemic samples such as blood reduces potential understanding of general immune processes. Since the interaction between miRNA and mRNA, as well as the identified therapeutic targets, need further experimental validation, the conclusions are derived from computational predictions.

## Ethical considerations

This study did not involve human participants or animals; and therefore ethical approval was not required.

## Data Availability

The datasets analyzed during the current study are publicly available in the NCBI Gene Expression Omnibus (GEO) database under accession number GSE207345 ( https://www.ncbi.nlm.nih.gov/geo/query/acc.cgi?acc=GSE207345). ^41^ Data are available under the terms of the Creative Commons Zero (No rights reserved) data waiver (CC0 1.0 Public domain dedication). Dataset citation: GSE207345. Single-cell RNA-seq analysis of pancreatic ductal adenocarcinoma and adjacent normal tissue. NCBI GEO. Available at: https://www.ncbi.nlm.nih.gov/geo/query/acc.cgi?acc=GSE207345. This study complied with the STROBE guidelines for observational studies.
