# Supplementary material for: Bioinformatics-based Investigation to Unveiling The miRNA-Immunity Axis in The Tumor Microenvironment of Pancreatic Cancer
Source: F1000Res. 2026 May 22;14:1404. Originally published 2025 Dec 16. [Version 3] doi: 10.12688/f1000research.172407.3 (PMC13291752; doi:10.12688/f1000research.172407.3)
Supplement: Peer review history

# Reviewer report: Reviewer response for version 3

**DOI:** 10.5256/f1000research.200514.r495985

**Published:** 2026-06-27

**Authors:** Jameel Rabee Al-Obaidi

**Affiliations:** 1 Universiti Pendidikan Sultan Idris, Tanjung Malim, Malaysia

General Assessment

 The authors have done a great deal to respond to the comments received in the previous round of review. The revised manuscript is much improved in scientific interpretation, clarity of presentation, description of methods, and discussion of limitations. The corrections for interpretation of pancreatic enzyme-related genes and the updated explanation for the enrichment of ductal cells have greatly enhanced the biological validity of the study. The manuscript now offers a coherent and comprehensive bioinformatics investigation of the potential role of miRNA-mediated regulation of memory CD4+ T cells in the pancreatic ductal adenocarcinoma (PDAC) tumor microenvironment.

 Single-cell transcriptomic analysis combined with miRNA expression profiling is an interesting strategy to develop hypotheses on the mechanisms of immune suppression in PDAC. Identification of candidate miRNAs that may target important immune-regulatory genes like SOCS3, NR4A2 and CXCR4 offers valuable information that could be used to direct future experimental validation studies.

 Strengths of the Manuscript

 1. Comprehensive Bioinformatics Framework

    The study is able to seamlessly combine scRNA-seq analysis, differential gene expression analysis, miRNA target prediction, and network analysis into one workflow. This multi-layered approach gives a comprehensive overview of immune regulation in PDAC.

 2. **Improved Biological Interpretation**

    The authors have made appropriate updates to previous interpretations concerning pancreatic enzyme gene expression and ductal cell abundance. These corrections enhance the biological rigor of the manuscript and show responsiveness to reviewers' comments.

 3. Identify study limitations

    The manuscript now explicitly states the limitations of using publicly available datasets, possible batch effects, and the lack of experimental validation. This will increase transparency and scientific balance.

 4. Focus on Memory CD4+ T Cells

    The focus on memory CD4+ T cells offers a new paradigm of the immune biology of PDAC and adds to the growing body of literature exploring T cell dysfunction in the tumor microenvironment.

 5. Improved Readability

    The text has been substantially rewritten to make it more readable, concise, and well organized than in earlier editions. The findings are now better integrated with the literature and there is some translation implications identified.

 Some minor suggestions for further improvement:

 The manuscript is now ready for indexing but there are a few minor improvements that can be made:

 The discussion sometimes makes inferences about biological mechanisms from computational predictions. The authors recognize this, but some statements might be modified to stress that these are observations at this stage and need to be confirmed by experiment.

 2. There are some repetitions in the Results section with regard to the use of figures. These descriptions could be condensed to make them easier to read.

 4. There should be a brief comment on whether the miRNAs identified in this manuscript have been previously reported in independent PDAC cohorts to increase the confidence in the biological relevance.

 4. There are some grammatical and stylistic inconsistencies in the manuscript. There are a few places that would benefit from an additional round of professional English editing to make it easier to read.

 Recommendation

 Overall, the authors have responded to the key concerns raised in the previous rounds of reviews. The study is well designed, the conclusions are generally warranted by the analyses presented, and the limitations are stated. Even though experimental validation is required to confirm the interactions between miRNA and mRNA, the current study of bioinformatics is still a valuable hypothesis-generating study.

 I feel the manuscript is acceptable for indexing with minor editing.

Is the work clearly and accurately presented and does it cite the current literature?

Yes

If applicable, is the statistical analysis and its interpretation appropriate?

Yes

Are all the source data underlying the results available to ensure full reproducibility?

No source data required

Is the study design appropriate and is the work technically sound?

Yes

Are the conclusions drawn adequately supported by the results?

Yes

Are sufficient details of methods and analysis provided to allow replication by others?

Yes

Reviewer Expertise:

Dr. Jameel R. Al-Obaidi is a Senior Lecturer at the Department of Biology, Faculty of Science and Mathematics, Universiti Pendidikan Sultan Idris (UPSI), Malaysia. A molecular biologist specializing in proteomics, his work focuses on the discovery and application of protein markers supported by AI-driven proteomics, metabolomics, and bioinformatics. He integrates experimental proteomics with advanced computational approaches to accelerate biomarker identification and functional analysis.

I confirm that I have read this submission and believe that I have an appropriate level of expertise to confirm that it is of an acceptable scientific standard, however I have significant reservations, as outlined above.

# Reviewer report: Reviewer response for version 3

**DOI:** 10.5256/f1000research.200514.r487578

**Published:** 2026-06-23

**Authors:** Fadehl Lafta

**Affiliations:** 1 Institute of Laser for Postgraduate Studies, University of Baghdad, Baghdad, Baghdad Governorate, Iraq; 2 Department of Biology, College of Science, University of Baghdad, Baghdad, Baghdad Governorate, Iraq

I have read the revised version of the manuscript “Bioinformatics-based Investigation to Unveiling The miRNA-Immunity Axis in The Tumor Microenvironment of Pancreatic Cancer” and I’m satisfied with the corrections made by the authors in response to the raised comments.

Is the work clearly and accurately presented and does it cite the current literature?

Yes

If applicable, is the statistical analysis and its interpretation appropriate?

I cannot comment. A qualified statistician is required.

Are all the source data underlying the results available to ensure full reproducibility?

Yes

Is the study design appropriate and is the work technically sound?

Yes

Are the conclusions drawn adequately supported by the results?

Yes

Are sufficient details of methods and analysis provided to allow replication by others?

Yes

Reviewer Expertise:

Molecular genetics/ DNA methylation

I confirm that I have read this submission and believe that I have an appropriate level of expertise to confirm that it is of an acceptable scientific standard.

## References

[1] : Integration of genome-level data to allow identification of subtype-specific vulnerability genes as novel therapeutic targets. *Oncogene* .2021;40(33) : 10.1038/s41388-021-01923-1 5213-5223 10.1038/s41388-021-01923-1 PMC837664534230614

# Reviewer report: Reviewer response for version 3

**DOI:** 10.5256/f1000research.200514.r488347

**Published:** 2026-06-23

**Authors:** Mahmoud Hamid Al-Fahdawi

**Affiliations:** 1 Al-Nahrain University, Baghdad, Iraq

This article presents a valuable bioinformatics analysis of the miRNA–immunity axis in pancreatic ductal adenocarcinoma. The study addresses an important topic and provides useful insights into the role of dysregulated miRNAs in modulating immune responses, particularly in memory CD4+ T cells.

 The manuscript is clearly written, supported by relevant literature, and employs appropriate analytical methods. The source data are publicly available, and the analyses are sufficiently described to support reproducibility.

 The results are clearly presented and support the conclusions. Overall, this is a scientifically sound and well-structured study that contributes to our understanding of immune regulation in PDAC. I recommend approval of the article.

Is the work clearly and accurately presented and does it cite the current literature?

Yes

If applicable, is the statistical analysis and its interpretation appropriate?

Yes

Are all the source data underlying the results available to ensure full reproducibility?

Yes

Is the study design appropriate and is the work technically sound?

Yes

Are the conclusions drawn adequately supported by the results?

Yes

Are sufficient details of methods and analysis provided to allow replication by others?

Yes

Reviewer Expertise:

Bioinformatics, Cancer Biology, Immunology, Single-Cell Transcriptomics

I confirm that I have read this submission and believe that I have an appropriate level of expertise to confirm that it is of an acceptable scientific standard.

# Reviewer report: Reviewer response for version 3

**DOI:** 10.5256/f1000research.200514.r487577

**Published:** 2026-06-03

**Authors:** Jörg Kleeff

**Affiliations:** 1 Martin-Luther-University Halle-Wittenberg, University Medical Center Halle, Halle, Germany

Although the authors have tried to address the questions/concerns of the reviewer, most of them not satisfactorily. Thus, my general concerns remain (see previous review). I still appreciate the effort of the authors; however, I cannot recommend the paper for publication in its present form.

Is the work clearly and accurately presented and does it cite the current literature?

Partly

If applicable, is the statistical analysis and its interpretation appropriate?

I cannot comment. A qualified statistician is required.

Are all the source data underlying the results available to ensure full reproducibility?

Yes

Is the study design appropriate and is the work technically sound?

Partly

Are the conclusions drawn adequately supported by the results?

Partly

Are sufficient details of methods and analysis provided to allow replication by others?

Yes

Reviewer Expertise:

-

I confirm that I have read this submission and believe that I have an appropriate level of expertise to state that I do not consider it to be of an acceptable scientific standard, for reasons outlined above.

# Reviewer report: Reviewer response for version 1

**DOI:** 10.5256/f1000research.190129.r444705

**Published:** 2026-04-25

**Authors:** Jörg Kleeff

**Affiliations:** 1 Martin-Luther-University Halle-Wittenberg, University Medical Center Halle, Halle, Germany

This study analyzed the tumor microenvironment of pancreatic ductal adenocarcinoma (PDAC) with a focus on memory CD4+ T cells and how their function is affected by microRNAs (miRNAs). Using single-cell RNA sequencing online data, the authors found that memory CD4+ T cells are more abundant in PDAC tumors than in normal pancreas tissue, but key immune and metabolic genes in these cells are suppressed. Bioinformatic analyses identified several overexpressed miRNAs—particularly hsa-miR-1207-5p, hsa-miR-6805-5p, and hsa-miR-149-3p—that likely target immune-related and pancreatic enzyme-associated genes. A core set of genes (CLPS, NR4A2, and SOCS3) was frequently targeted, suggesting a role in immune evasion. Overall, the findings indicate that although memory CD4+ T cells are present in PDAC, miRNA-driven gene repression limits their activity, highlighting miRNAs as potential therapeutic targets to restore anti-tumor immune responses.

 The topic of this manuscript—namely, the tumor-suppressive microenvironment in pancreatic cancer—is timely, relevant, and worthy of investigation. The authors employ publicly available datasets to explore this question, and the statistical analyses appear sound and appropriate. However, a major limitation of the study is its exclusive reliance on online databases, without validation in independent patient cohorts or experimental confirmation, which restricts the overall robustness of the conclusions. In addition, the level of novelty is limited, as several of the presented findings have already been reported in previous studies. Consequently, while the analysis is methodologically solid, its impact is constrained by the lack of external validation and incremental novelty.

 There are also conceptual misconceptions in the interpretation of some results. First, the authors state that pancreatic enzyme genes are markedly repressed. However, this observation does not reflect true gene repression or regulatory mechanisms. Instead, it is a consequence of tissue composition: during pancreatic cancer progression, normal pancreatic tissue—predominantly composed of acinar cells—is replaced by cancer cells and a pronounced stromal reaction. Consequently, tumor tissue samples naturally contain few or no acinar cells, and reduced expression of acinar enzyme genes is therefore expected. The manuscript and the subsequent discussion should be carefully revised to reflect this distinction.

 In a similar vein, the authors suggest that an increased proportion of ductal cells may represent a compensatory or pathological response to tumor development. This interpretation is misleading. Pancreatic ductal adenocarcinoma inherently expresses ductal cell markers; thus, bioinformatic classification of a substantial fraction of tumor cells as having a ductal phenotype likely reflects malignant cells rather than normal ductal cells. This issue should also be addressed and corrected throughout the manuscript.

 Furthermore, the analysis consistently compares tumor tissue with normal pancreatic tissue. While this is informative, it would have been particularly interesting to compare the immune microenvironment across different pancreatic cancer subtypes, such as classical versus squamous PDAC, which could provide more biologically relevant insights.

 As minor points, the Results section concludes with only very brief summary statements (e.g., “Donor-based clustering and pancreatic cell type annotation are shown in ( Figure 2)”; or “Immune cell enrichment between PDAC and healthy tissues is presented in ( Figure 3)”), which do not adequately synthesize the findings. A more integrative summary would strengthen this section. Additionally, the manuscript contains some awkward expressions e.g. “…talked in language that was even more humanlike”.

Is the work clearly and accurately presented and does it cite the current literature?

Partly

If applicable, is the statistical analysis and its interpretation appropriate?

I cannot comment. A qualified statistician is required.

Are all the source data underlying the results available to ensure full reproducibility?

Yes

Is the study design appropriate and is the work technically sound?

Partly

Are the conclusions drawn adequately supported by the results?

Partly

Are sufficient details of methods and analysis provided to allow replication by others?

Yes

Reviewer Expertise:

NA

I confirm that I have read this submission and believe that I have an appropriate level of expertise to confirm that it is of an acceptable scientific standard, however I have significant reservations, as outlined above.

# Reviewer report: Reviewer response for version 1

**DOI:** 10.5256/f1000research.190129.r448999

**Published:** 2026-01-22

**Authors:** Fadehl Lafta

**Affiliations:** 1 Institute of Laser for Postgraduate Studies, University of Baghdad, Baghdad, Baghdad Governorate, Iraq; 2 Department of Biology, College of Science, University of Baghdad, Baghdad, Baghdad Governorate, Iraq

The submitted manuscript provides a key insight to the miRNA alterations in the context of pancreatic cancer that could be utilized in the development of targeted therapies.

 The study is well-designed and drafted; however, addressing the following point could improve the manuscript quality.

1. How memory CD4 T cells contribute to the altered PDAC tumor microenvironment.
2. Gene names should be written in an Italic style.
3. Some abbreviations need to be written in full terms.

Is the work clearly and accurately presented and does it cite the current literature?

Yes

If applicable, is the statistical analysis and its interpretation appropriate?

I cannot comment. A qualified statistician is required.

Are all the source data underlying the results available to ensure full reproducibility?

Yes

Is the study design appropriate and is the work technically sound?

Yes

Are the conclusions drawn adequately supported by the results?

Yes

Are sufficient details of methods and analysis provided to allow replication by others?

Yes

Reviewer Expertise:

Cancer molecular genetics

I confirm that I have read this submission and believe that I have an appropriate level of expertise to confirm that it is of an acceptable scientific standard, however I have significant reservations, as outlined above.

# Response

**Published:** 2026-01-23

**Authors:** Mustafa Fahad

**Affiliations:** Center of Desert Studies, University of Anbar, Ramadi, Al Anbar Governorate, Iraq

We thank the reviewer for the constructive comments. We will clarify the contribution of memory CD4+ T cells to PDAC TME and adjust gene formatting and abbreviations accordingly in the revised version.”

# Response

**Published:** 2026-06-04

**Authors:** Fadhel Lafta

**Affiliations:** Biological and Medical Branch, University of Baghdad Institute of Laser for Postgraduate Studies, Baghdad, Baghdad Governorate, Iraq

I accept the User Comment Terms and Conditions.
